# Dorsoventral and Proximodistal Hippocampal Processing Account for the Influences of Sleep and Context on Memory (Re)consolidation: A Connectionist Model

**DOI:** 10.1155/2017/8091780

**Published:** 2017-07-03

**Authors:** Justin Lines, Kelsey Nation, Jean-Marc Fellous

**Affiliations:** ^1^Department of Psychology, University of Arizona, Tucson, AZ 85721, USA; ^2^Neuroscience Graduate Interdisciplinary Program, University of Arizona, Tucson, AZ 85721, USA; ^3^Program in Applied Mathematics, University of Arizona, Tucson, AZ 85721, USA

## Abstract

The context in which learning occurs is sufficient to reconsolidate stored memories and neuronal reactivation may be crucial to memory consolidation during sleep. The mechanisms of context-dependent and sleep-dependent memory (re)consolidation are unknown but involve the hippocampus. We simulated memory (re)consolidation using a connectionist model of the hippocampus that explicitly accounted for its dorsoventral organization and for CA1 proximodistal processing. Replicating human and rodent (re)consolidation studies yielded the following results. (1) Semantic overlap between memory items and extraneous learning was necessary to explain experimental data and depended crucially on the recurrent networks of dorsal but not ventral CA3. (2) Stimulus-free, sleep-induced internal reactivations of memory patterns produced heterogeneous recruitment of memory items and protected memories from subsequent interference. These simulations further suggested that the decrease in memory resilience when subjects were not allowed to sleep following learning was primarily due to extraneous learning. (3) Partial exposure to the learning context during simulated sleep (i.e., targeted memory reactivation) uniformly increased memory item reactivation and enhanced subsequent recall. Altogether, these results show that the dorsoventral and proximodistal organization of the hippocampus may be important components of the neural mechanisms for context-based and sleep-based memory (re)consolidations.

## 1. Introduction

The memory formation process can be decomposed into acquisition, consolidation, and reconsolidation phases in which information is gained, stored, and modified, respectively [1, 2]. Consolidation involves the integration of a new memory into an existing memory network; however, that memory can become labile and may be updated through one of several episodes or reconsolidations [3–5]. During this process, a memory item is linked with both newly formed and previously stored memory items [6, 7]. Though the mechanisms of reconsolidation are still unclear, behavioral studies in humans and rats have shown that passive reexposure to a past context is sufficient to reactivate and alter a learned set of objects that was learned in that context [8–11].

Memories can be reconsolidated through associational reactivation, and memories have also been shown to replay during sleep [12–14]. Memory replay has been proposed as the major neural mechanism supporting the consolidation of recently formed memories [15–17]. Experimental studies suggest an active consolidation process during sleep both behaviorally and at the cellular level [18–20]. Interrupting sleep-induced memory replay is detrimental to consolidation and to subsequent recall performance [21]. Conversely, targeted memory reactivations during sleep by reintroducing an odor or sound associated with a behavioral task during sleep have been shown to enhance memory reactivation leading to increased behavioral performance the next day [22–24]. These studies have provided behavioral data that can be used to validate mechanistic models of the (re)consolidation process.

The “trisynaptic circuit” of the hippocampal formation is believed to be central to memory processing [25, 26]. Input to the hippocampus originates from superficial layers of the entorhinal cortices [27, 28]. Contextual information from the parietal “where” pathway enters the hippocampus through the medial entorhinal cortex (MEC), which, with the discovery of spatially selective grid and boundary cells, has been shown to depend on an animal's surroundings [29, 30]. Complementary to this contextual input, the lateral entorhinal cortex (LEC) has been shown to provide the hippocampus with nonspatial, object-related sensory “what” information from temporal lobe neurons [31, 32]. Inputs from the entorhinal cortex into the hippocampus are orthogonalized throughout the relatively large dentate gyrus (DG) before reaching area CA3 [33, 34]. Neurons within CA3 have highly recurrent connectivity and have been suggested to autocomplete partial signals and form memory associations before being sent to CA1 [35–37]. It has been further suggested that subregions of CA1 more proximal to CA3 compare this associational information with unprocessed contextual information from the MEC, whereas distal regions integrate CA3 output with direct object information from the LEC [38, 39]. Thus, memories and the context in which they occur are processed in an interacting fashion in the trisynaptic circuit of the DG, CA3, and CA1.

Experimental studies have also suggested a functional distinction along the dorsoventral axis of the hippocampus [40–42]. The dorsal hippocampus is necessary for spatial navigation because it receives more inputs from sensory cortices than the ventral hippocampus. Output projections from the dorsal and ventral hippocampus also differ, but the organization of the trisynaptic network is conserved along the dorsoventral axis. Functionally, place cells fire over a larger area of space ventrally, suggesting that the environment is encoded at different scales along the dorsoventral axis. Additionally, patterns formed in the dorsal CA3 have been shown to be biased towards object representations, while ventral CA3 place cells encode contexts [43, 44]. Computational models have been used to understand place field size variability and functional roles in spatial navigation along the dorsoventral axis [45–47], but no computational models have been used to investigate the dynamics of the memory formation process stemming from this functional segregation.

The dorsoventral organization of the hippocampus and that of the proximodistal axis of the CA1 region have been largely absent in past computational models constructed from hippocampal anatomy [48], making it difficult for these models to separate the complex dynamics of object and context associations. Current models simulating contextual based object reconsolidation tackle this problem via conceptual modeling of memory dynamics, using the temporal evolution of context or set associations [49, 50]. In addition, simulations of sleep processes have focused mainly on memory reactivation [51, 52] and not consolidation during sleep.

Previous work has shown that the spatial context had an important influence on the manner with which two memories learned at different times are stored and recalled [8–11]. These experimental results, and many others, make the strong, yet reasonable, assumption that the memory items learned are independent from each other. Experimentally testing the amount of semantic overlap between items is notoriously difficult and subject to a large amount of individual differences. However, computational models allow for a systematic study of the influence of this overlap on recall. Furthermore, all experimental paradigms to date involve an offline period during which subjects undergo a “break” or “wait” period during which their activity is uncontrolled and in many cases not monitored. Subjects undoubtedly acquire new memories during that time, some of which may significantly interfere with the items that were part of the experiments. The influence of this new learning on the recall performance of the subject is unknown. Again, computational models allow for a systematic study of this influence by controlling the amount of interfering learning.

In this study, we use a connectionist model highlighting the functional difference between the dorsal and ventral levels of the hippocampus and the difference between proximal and distal CA1 computations to study the associational and spontaneous memory reactivations leading to (re)consolidation.

## 2. Materials and Methods

### 2.1. Model Architecture

**Figure 1:**
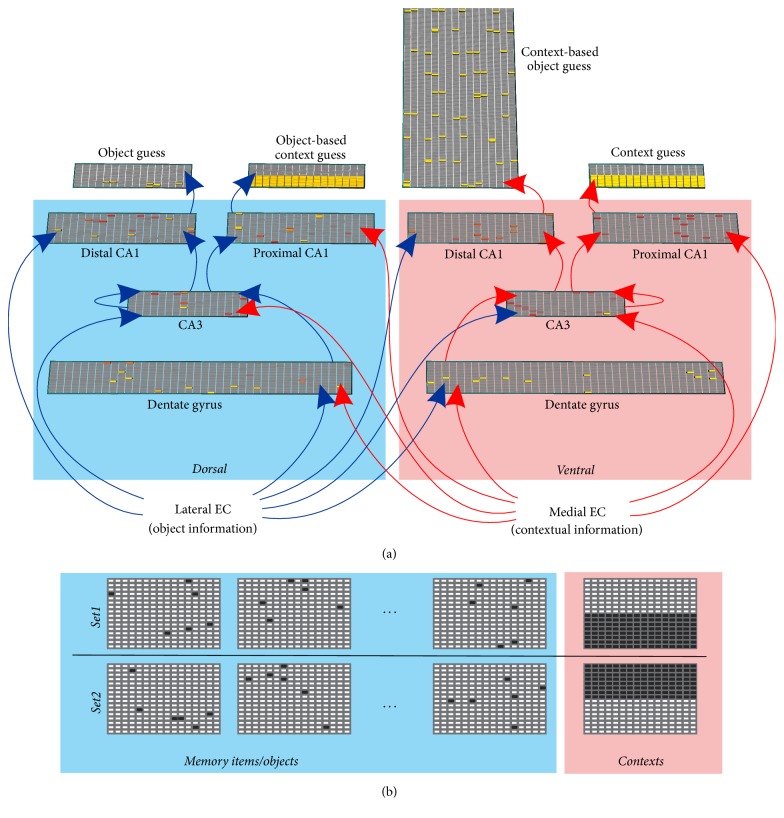
Model architecture. (a) Inputs to the model are segregated into two pathways with item information entering the dorsal hippocampus and context information entering the ventral hippocampus. Both information types interact and influence each other to yield 4 outputs. Element color denotes level of activity with yellow > red > grey. (b) Different inputs are represented by different subsets of active EC neurons (black squares). For simplicity contextual inputs are represented as nonoverlapping blocks.

The network structure we present here was created from anatomical and functional studies, compared to other architectures, and selected for its ability to form object-context associations that are robust in the face of 5% parameter variations [46]. The model was implemented using the Emergent simulator [53]. The network included interacting dorsal and ventral divisions at all hippocampal subsections and we further segregated the CA1 along the proximodistal axis, as suggested by experimental work (Figure 1(a)) [39]. Inputs were represented by patterns of activation in the lateral entorhinal cortex (LEC, layer size 16 × 16) and medial entorhinal cortex (MEC, layer size 16 × 16, Figure 1(b)). These layers sent outputs to dorsal and ventral dentate gyrus (DG, 2 layers, dorsal and ventral, size 20 × 40 each), CA3 (2 layers size 16 × 16 each), and CA1 (4 layers size 20 × 20 each, Table 1). Projections from the DG reached CA3 which in turn sent outputs to CA1. The two CA3 layers included recurrent all-to-all connections to themselves (Table 2). Each CA1 was further partitioned into distal (layer size 20 × 20) and proximal (layer size 20 × 20) subdivisions that received monosynaptic inputs from the LEC and MEC, respectively. The choice of the layer sizes was motivated by computational considerations, to yield enough combinatorial choices for the various manipulations used (e.g., noise variation, semantic overlap) in the simulations. Of the eight intrahippocampal layers, the dorsal-distal and proximal-ventral CA1 layers were the only ones not receiving monosynaptic inputs from the opposite streams (the dorsal-distal layer does not have monosynaptic input from the ventral stream and the proximal-ventral layer does not have monosynaptic input from the dorsal stream). Since, by design, the dorsal stream was dominated by object information and the ventral stream was dominated by contextual information, we labeled the output of dorsal-distal and proximal-ventral layers “object guess” and “context guess,” respectively. The dorsal-proximal CA1 layer receives monosynaptic inputs from the context-dominated stream (ventral, MEC inputs) and should therefore carry context information. However, because it is polysynaptically biased by object information (dorsal/LEC stream), this contextual information is in part based on the nature of the object. We therefore labeled the output of the dorsal-proximal CA1 “object-based, context guess.” Similarly, the output of the ventral-distal CA1 is labeled “context-based object guess.” Because there is only one context and multiple objects, the size of the object-based context guess layer is identical to that of the context representation, while that of the context-based object guess is scaled to the size of the possible number of objects present per context.

### 2.2. Simulations

Prior to simulations, all synaptic connection weights were initialized at random. The model was trained in discrete epochs, whereby at the end of an epoch synaptic weights between nodes were updated in an error driven manner using the generalized recirculation algorithm (GeneRec) of the Leabra model system [53–55]. Object and contextual information used for training were represented by binary 16 × 16 matrices introduced to the network through the LEC and MEC, respectively (Figure 1(b)).

**Figure 2:**
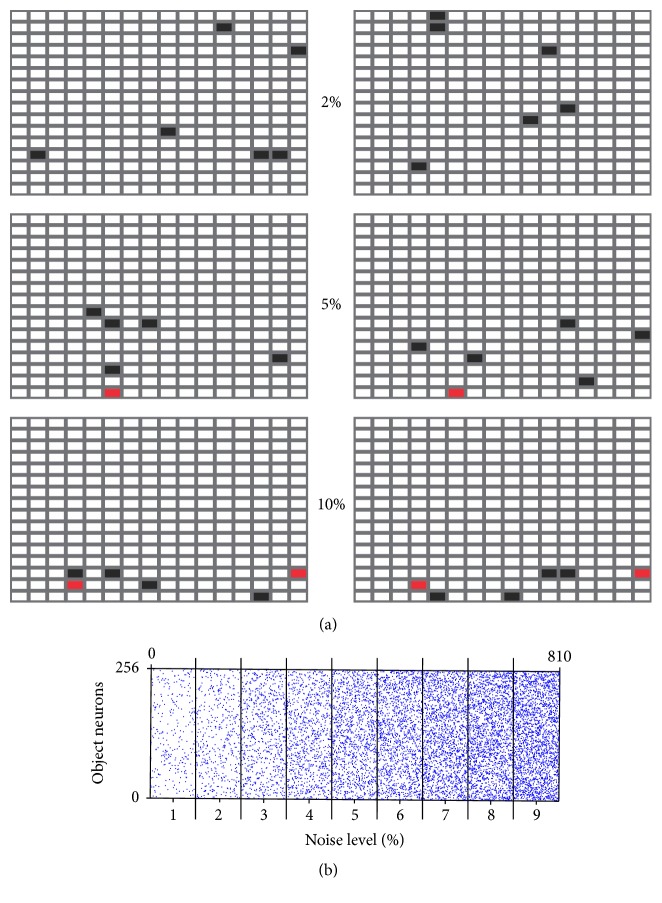
Input variations in object representations. (a) Semantic overlap between 2 objects (left and right columns) consists in activating identical neurons in the two objects layers (black squares with red squares overlapping). Examples are given for 2, 5, and 10% overlaps. (b) Representation noise is introduced by randomly activating neurons each time an item is presented. For clarity, objects are shown as linear arrays of 256 neurons. 90 objects are presented for each noise level.

In some simulations, we manipulated the similarity between different objects, to capture the likely subjective semantic links between the stimuli presented experimentally in humans (e.g., doll and fire-truck are both “toys” [8]) or in rats (e.g., set items are rewarded by the same reward delivery system [10]). Object overlap was implemented by limiting the region of potential activity within the 16 × 16 matrix (Figure 2(a), top: no limits, only a subset of the neurons of the lower half of the matrix was used, bottom: only the lower third). Overlap in a network layer requires that individual representations share active elements. Constraining the active elements to a subset of the neurons forces the entries to recapitulate this patterning. Since input layers into the network include neither lateral inhibition nor connections that rely on spatial arrangement, performing simulations in this manner is identical to distributing this overlapped subset of active elements throughout the input matrices. The overlap between two objects was assessed post hoc using (1), measuring the similarity between two object representations. During testing, an object was considered recalled correctly if the object guess output had a similarity of 0.5 or more with any of the input objects presented during that test.

In the reactivation experiments, noise inputs used to reactivate previously learned object memories were constructed by pseudo-randomly activating 1–9% of the object neurons, ensuring that the total number of active neurons was kept constant (Figure 2(b)).

(1) $$\begin{array}{c} similarity=\frac{\left|A\cdot B\right|}{\sqrt{\left|A\right|\left|B\right|}}. \end{array}$$

### 2.3. Reconsolidation Simulations

**Figure 3:**
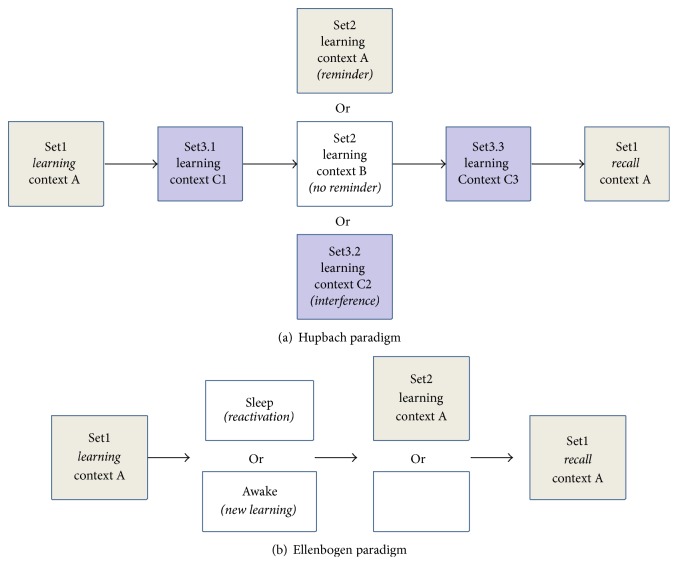
Simulation paradigms. (a) Paradigm models on Hupbach et al. 2007. Intervening learning (Set3.x) was added in the break periods to account for the cognitive activity of subjects during the 48 h experimental gaps they were given. (b) Paradigm modeled on Ellenbogen et al. 2006.

We first investigated context-based object memory reconsolidation as was done in human and rodent studies [8–11]. In these experiments, subjects learned a first set of items in spatial context A (Set1: 20 objects for humans, 3 out of 8 feeder locations for rats, Figure 3(a)). After a fixed amount of time (break), a second set of items were presented for learning (Set2), in either the same context (reminder group) or a different context (no-reminder group). A separate group was not presented with any new learning (interference control). After a fixed amount of time, subjects were then asked to recall the first set of items (Set1 recall). In the following, we focus on the human version of the task. To model learning, we trained the network on a set of 20 objects (pseudo-randomly presented) in a given context until the error between the network input and output was below 15% within 4 epochs of training. To account for the fact that human subjects likely perform additional, possibly interfering, learning in the break periods, the model also performed additional item learning after Set1 or Set2 learning in unique contexts different from A and B made up of randomly distributed elements around the entire context matrix. The objects learned (Set3.x) were different from that of Set1 and Set2 but were allowed to have an average overlap of 2.5 +/− 0.5% with sets 1 and 2. As in the human experiments, a control simulation was also performed where the model skipped Set2 learning and was only trained on a new set of objects in a unique context* (interference)*. Object guess outputs that were similar to an object from Set1 were labeled as correctly recalled, while objects similar to objects from Set2 were labeled as intrusions from Set2. We fit the performance of the model to experimental data by varying both the average overlap between the 40 objects comprising Set1 and Set2 as well as the combined sizes of intermediate Set3.x learning sets. To compare human and rodent experimental (exp) results with our simulations (sim) we minimized the distance function shown in (2) which simultaneously accounts for the interference (int), reminder (rem), and no-reminder (norem) conditions.

(2) $$\begin{array}{c} dist\left(\;i\;\right)=\sqrt{{\left({int}_{exp}\left(i\right)-{int}_{sim}\left(i\right)\right)}^{2}+{\left({rem}_{exp}\left(i\right)-{rem}_{sim}\left(i\right)\right)}^{2}+{\left({norem}_{exp}\left(i\right)-{norem}_{sim}\left(i\right)\right)}^{2}}. \end{array}$$

To determine the extent to which the recurrent network of CA3 is involved, we selectively reduced the number of cells composing the dorsal, ventral, or entire CA3 by 25%, 50%, 75%, and 100% of its nominal size. These adjusted models were then used to simulate the context-based object memory reconsolidation paradigm.

### 2.4. Sleep Simulations

To investigate the role of sleep in memory consolidation, we trained the model using a different experimental paradigm [18, 56]. After learning a set of 20 objects, memory reactivation was induced by testing the model on 810 object-context pairs composed of 1–9% random activity (90 object-context pairs per noise level, Figure 2(b)). The network was tested with noise inputs, but with synaptic plasticity disabled, to allow memory reactivations to be spontaneously recalled based on the synaptic weights established during previous object-context pair learning. To simulate reactivation during sleep, objects for which output layers were at least 60% similar to one of the previously learned objects were “fed back” into the network for 3 epochs. These 3 reverberations simulated the process of reactivation.

Using this protocol of sleep consolidation, we simulated human behavioral results [18] as follows: the network was trained on a set of 20 objects (here, an object is akin to the “word pair” used in the experiments) to 15% error within a given context A. These simulations were followed by either a sleep period (reactivation, as described above) or a “wake” period, implemented as in the reconsolidation experiments by additional learning. In these simulations, the model was required to learn 3 sets of interfering memory items (20 items per set) in 3 different contexts. In the human experiments, memory resilience was challenged by having subjects learn a new set of objects upon waking, prior to recalling the original object set. To investigate the effect of the sleep and wake simulations on memory resilience from subsequent interference, we performed additional training on a new set of 20 objects (Word pairs 2) before recalling the original set (Figure 3(b)). Finally, to simulate sleep as a passive mechanism that merely protected memories from interfering sensory inputs, we also performed simulations without sleep or wake epochs.

### 2.5. Memory Enhancement during Sleep

We conducted an additional set of simulations to test the possibility that targeted memory reactivations during sleep may enhance memory consolidation [22–24, 57, 58]. In the experimental study, subjects exposed to the same odor during learning and subsequent sleep showed increased performance in memory recall upon waking. In our simulations, the odor was represented by a partial contextual element, as has been previously suggested [59]. To model the human studies, the network was trained on a set of 20 objects in a given context before performing the sleep simulation with a partial representation of the context in half of the inputs to the MEC. A partial context was represented by allowing every active entry of the original context to have a pseudo-random value of 0-1 in addition to the 1–9% noise fed into the network during memory replay. 

### 2.6. Statistical Analyses

Two-sampled unpaired *t*-tests were used to test significance on data simulated using the model. Significance is indicated as ^*∗*^*p* < 0.05, ^*∗∗*^*p* < 0.01, and ^*∗∗∗*^*p* < 0.001.

## 3. Results

**Figure 4:**
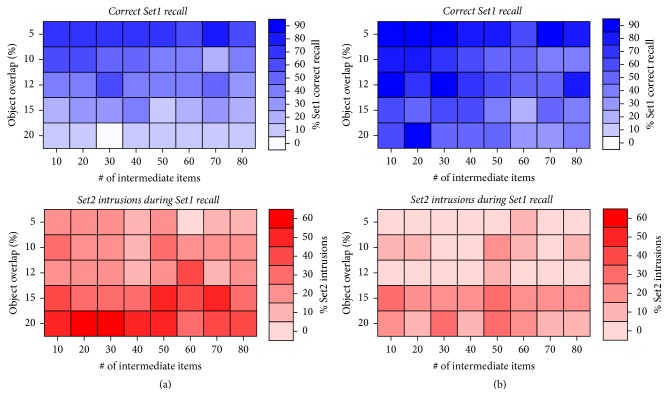
Effect of varying semantic overlap and amount of intermediate learning (i.e., Set3) on reconsolidation performance. (a) Same context condition. (b) Different contexts condition.

Our simulations allow for an investigation of semantic object overlap as well as the effect of interfering learning on memory stability.  Figure 4 shows the result of systematically varying the amount of semantic overlap among objects (*y*-axis) and the amount of additional, unrelated, learning during the wait/break periods (*x*-axis) on the amount of correctly recalled Set1 items (top) and the amount of context-induced intrusions of Set2 items into Set1 recall (bottom). In both context conditions, the large majority of Set1 items were correctly recalled (Figures 4(a) and 4(b), top), although learning Set1 in a different context than Set2 (different contexts condition, Figure 4(b) top) yielded better performance. Note that the parameter variations did not yield a strictly smooth change in recall (or intrusion, see below) measures due to the discrete sampling of nonlinear interactions between overlap (continuous variable) and number of intermediate items (discrete variable). Overall, semantic overlap was found to be inversely proportional to Set1 recall, and increasing the number of intermediate items learned had little to no effect on Set1 recall. In the condition where both sets were learned in the same context (same context condition, Figure 4(a), bottom), the overall amount of intrusions was significantly larger than in the different contexts condition (Figure 4(b), bottom), as observed experimentally. In general, larger semantic overlap of objects yielded less correct recall and larger amounts of intrusions. In contrast, increasing the number of additional items learned while keeping overlap constant did not significantly change the amount of correct recall or intrusions (Figure 4(a) bottom).

**Figure 5:**
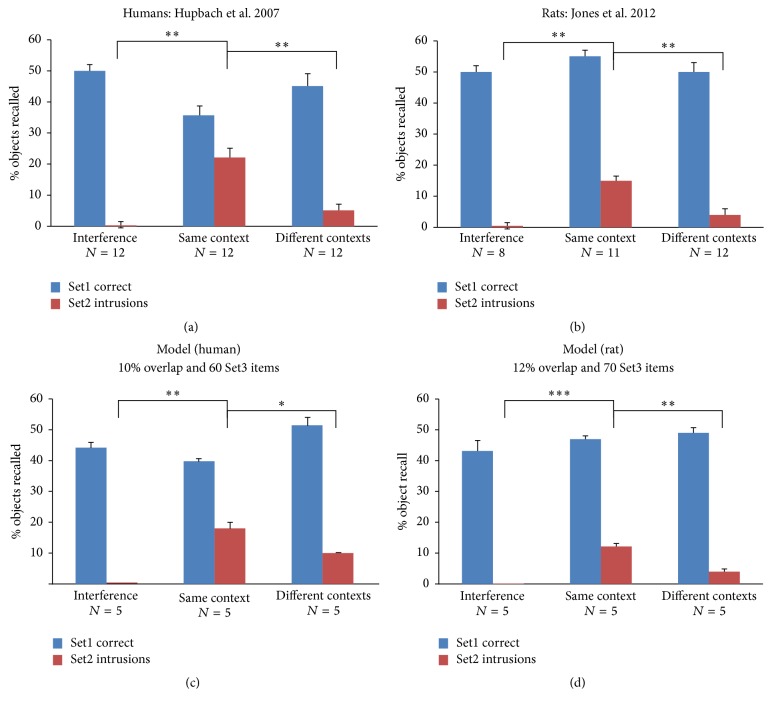
Fit to experimental data. (a) Behavioral data obtained using human subjects (adapted from Hupbach et al. 2007). (b) Behavioral data obtained using rats (adapted from Jones et al. 2012). (c) Best model fit to human data. (d) Best model fit to rat data. (c and d) The amount of representation (semantic) overlap and the number of intervening items learned during the wait/break periods were independently varied. Error bars are standard error of the mean. ^*∗*^*p* < 0.05, ^*∗∗*^*p* < 0.01, and ^*∗∗∗*^*p* < 0.001.

Experimental studies in humans (Figure 5(a)) and rats (Figure 5(b)) have shown that the overall amount of correctly recalled Set1 items was similar in both context conditions and during the interference control (blue bars, Figures 5(a) and 5(b)). However, a clear difference was found in the amount of intrusions produced during recall. Intrusions were significantly more numerous if Set2 items were learned in the same context as Set1 items (red bars, Figure 5(a)). Using the simulations shown in Figure 4, only varying the amount of semantic overlap and intervening learning, we found that best fits to the experimental data were slightly different for humans and rats. We found that 10% object overlap and 60 objects of intervening learning (Set3) were the best fit to human data (Figure 5(c)) and that 12% object overlap and 70 intervening learned objects were the best fit to rodent experimental data (Figure 5(d)).

**Figure 6:**
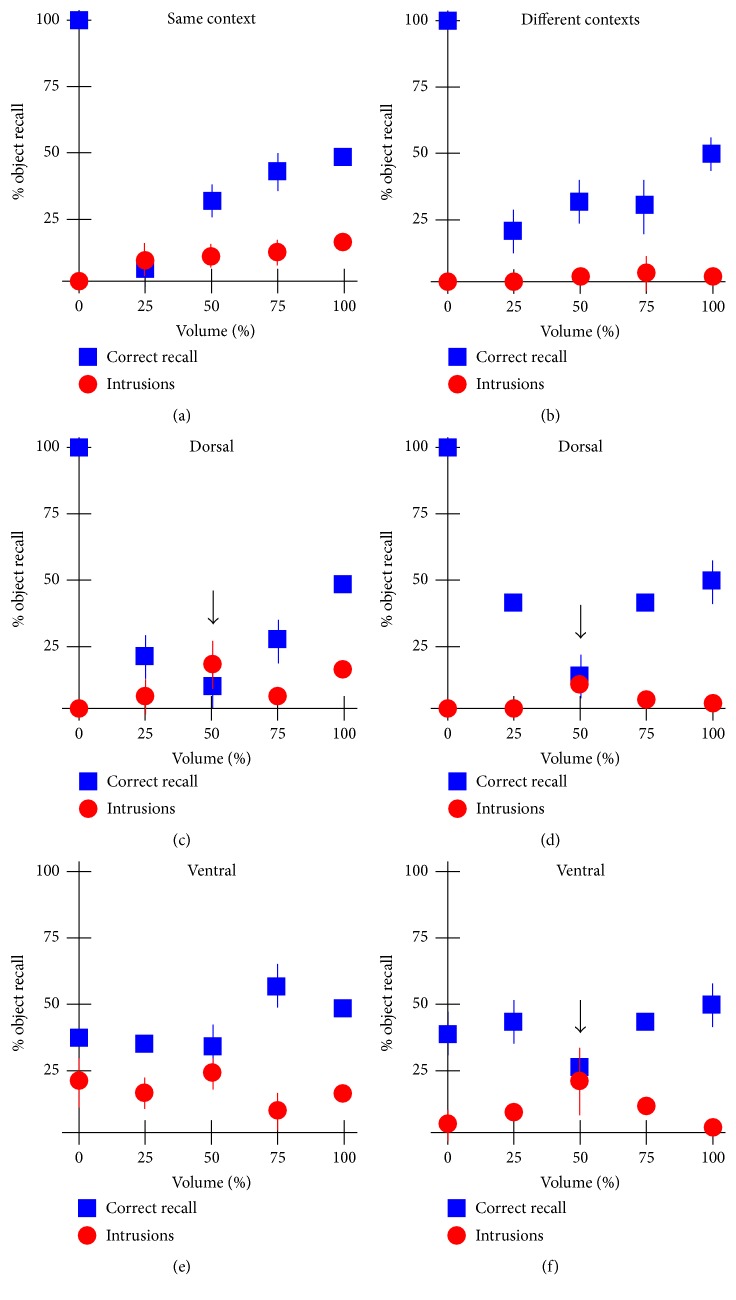
Role of CA3 in memory consolidation. Simulations were performed separately for the same (a, c, e) and different (b, d, f) context conditions. (a and b) show the effect of inactivating random subset of CA3 neurons (both dorsal and ventral) on the fraction of Set1 items correctly recalled and on the fraction of Set2 intrusions. Panels (c and d) show the effect of selective dorsal inactivation and panels (e and f) show the effect of selective ventral inactivation. Error bars are standard deviation.

Because the heavily recurrent networks of CA3 have long been known to perform functions such as pattern completion [36, 37], we studied their influence on memory reconsolidation by artificially inactivating a random subset of CA3 neurons. Taking the parameters derived to match the rodent data above, without inactivation (100% volume), the amount of correctly recalled Set1 items and the amount of intrusions match the behavioral data for the same and different contexts (Figures 6(a) and 6(b), 100% volume data points). Increasing the levels of inactivation of the entire structure yielded a progressive decrease in the amount of correctly recalled items (blue squares, decreasing volume), with little (same context, Figure 6(a)) or no effect (different contexts, Figure 6(b)) on intrusions. By design (Figure 1), CA3 receives a mixture of object and context information. Next we studied the effect of selectively inactivating the dorsal or ventral CA3. Selective dorsal CA3 inactivation had a biphasic effect on Set1 correct recall. Irrespective of context, behavioral performance worsened until about 50% inactivation at which point there was about the same amount of correct recall as intrusions (Figures 6(c) and 6(d), arrows). A further reduction in CA3 neurons restored correct Set1 recall performance somewhat, leaving intrusions low. Interestingly, inactivation of the ventral CA3 had comparatively little effect on Set1 correct recall, with a small dip in performance at 50% inactivation in the different context condition only (Figure 6(f) arrow). Set2 intrusions remained similar to that in the intact (100% condition) at all other levels of inactivation. Interestingly, a total inactivation of CA3 or of just dorsal CA3 yielded perfect recall and negligible intrusions in both context conditions, suggesting that the complex recurrent networks of dorsal CA3 may be responsible for the effect observed (lower correct Set1 performance and context-dependent intrusions).

**Figure 7:**
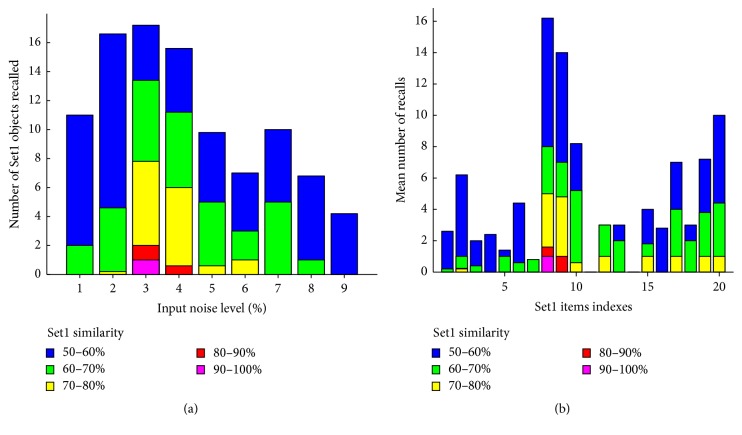
Simulations of replay during sleep. (a) Response of the network to noisy inputs of different levels. After each presentation, the object guess output layer is correlated with each of the Set1 items, and the highest correlation above 50% is tallied. (b) Same data as in (a), plotted per Set1 memory item.

Sleep is characterized by spontaneously occurring episodes of reactivation during which the neural representations of recently acquired memory items replay. These replay events are thought to be supported by various oscillatory mechanisms such as hippocampal sharp waves and corticothalamic spindles, though the exact manner in which these oscillations constrain replay is not yet clear. It is also not clear which memory items are replayed and for what reason. As a first step, we used the model to induce memory reactivation during sleep. We trained the network on a set of 20 objects with 10% average overlap. To simulate memory replay during sleep, we first disabled synaptic plasticity and let the network spontaneously respond to varying amount of object and context noise applied to its input layers. Because the synaptic connections were established by the learning of Set1, we hypothesized that the network would respond nonrandomly in a manner that would reflect Set1 items. Differing amounts of input noise produced object guesses that presented various amounts of similarity with Set1 objects (Figure 7(a)). The graph shows that small (1-2%) or large (7–9%) amounts of input noise were not able to produce outputs with similarity greater than 60% with Set1 (blue and green bars only). Intermediate noise levels (3–6%) were able to increase the similarity, with up to 90% similarity with Set1 for 3% noise levels. 3% noise was optimal and was commensurate with the amount of activity the LEC received during object learning (6/256 = 2.4%); however, these stochastic inputs were unrelated to the objects replayed (object/noise similarity = 3.45 ± 0.11%). Averaging across those bins revealed that Set1 items were replayed unevenly with some items not being replayed at all (e.g., items 11 and 13, Figure 7(b)) and others being replayed much more than the others (e.g., items 8 and 9, Figure 7(b)). Testing the hypothesis that this uneven object replay was related to initial object learning, we noted that the similarity of objects recalled after training was linearly correlated to their similarity during replay (*r* = 0.5678). Control simulations without Set1 learning showed no object guess outputs with a similarity greater than 50% (not shown).

**Figure 8:**
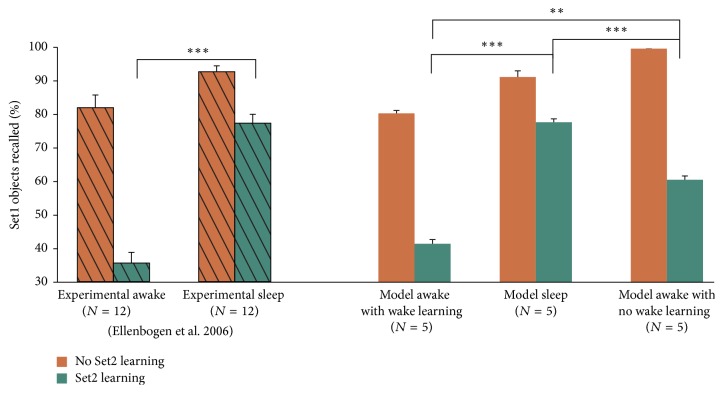
Sleep after learning protects memory. Left: experimental data adapted from Ellenbogen et al. 2006. Right: modeling results. *N* represents the number of subjects (experimental) or number of independent simulations (model). Error bars are standard error of the mean. ^*∗*^*p* < 0.05, ^*∗∗*^*p* < 0.01, and ^*∗∗∗*^*p* < 0.001.

To investigate the role of memory reactivation on memory consolidation during sleep, we developed a protocol to train the network on reactivated items. We first trained the network on 20 Set1 items with 10% average overlap. After training, synaptic plasticity was disabled (frozen synaptic weights, as above) and 810 inputs comprised of 1–9% random noise (90 object-context pairs per percentage of noise, Figure 2(b)) were input to the network. If an object guess had a similarity of at least 60% with a Set1 item, then the object guess and context guess were fed back into the LEC and MEC, respectively, and synaptic plasticity was allowed to occur for 3 epochs (i.e., reactivation-driven synaptic modifications). In order to test our proposed sleep-induced memory consolidation protocol, we simulated the experiments of Ellenbogen et al. (2006) in which sleep was found to protect newly acquired memory from interference due to postsleep learning (Figure 3(b)).  Figure 8 (left) summarizes the experimental results. Without additional Set2 learning, subjects could recall Set1 items very well, whether they were allowed to sleep or not before recall (orange hashed bars, Figure 8), with a small marginally significant enhancement of recall if sleep was allowed (0.5 < *p* < 0.1, not shown). If Set2 learning was introduced, however, a significant difference was found. Subjects that were not allowed to sleep recalled less than 40% of the Set1 items, whereas subjects that were allowed to sleep could recall almost 80% of the Set1 objects (green hashed bars, Figure 8). Using the model as it was tuned in the previous simulations, and without any new modifications, we could closely reproduce the experimental data under the assumption that subjects kept awake were in fact undergoing additional learning (“model awake with awake learning,” Figure 8). Removing this additional learning reduced the goodness of fit and produced larger no-set2 learning performance and lower Set2 learning performance than experimentally observed (Figure 8, right most bars). This result suggests that the model architecture and replay protocol are sufficient to capture the performance features of this experiment and that part of the reason why performance of the awake group is so low may rely on the fact that subjects undergo uncontrolled/unmonitored learning that interferes with the storage of the experimental memory items. In the experiments and the model, sleep did not increase recall but increased resilience to additional interference learning (Set2).

**Figure 9:**
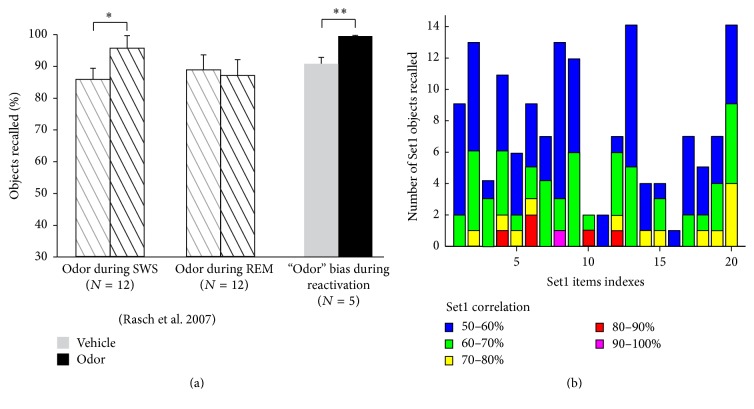
Context-biases replay. (a) Experimental data of Rasch et al. 2007 show that presentation of the odor with which Set1 items were learned produced a better recall if the odor was presented during SWS but not during REM sleep (hashed bars). Our model correctly reproduced the SWS data (grey and black bars). (b) All Set1 items were significantly replayed during simulated sleep. Error bars are standard error of the mean. ^*∗*^*p* < 0.05, ^*∗∗*^*p* < 0.01, and ^*∗∗∗*^*p* < 0.001.

**Figure 10:**
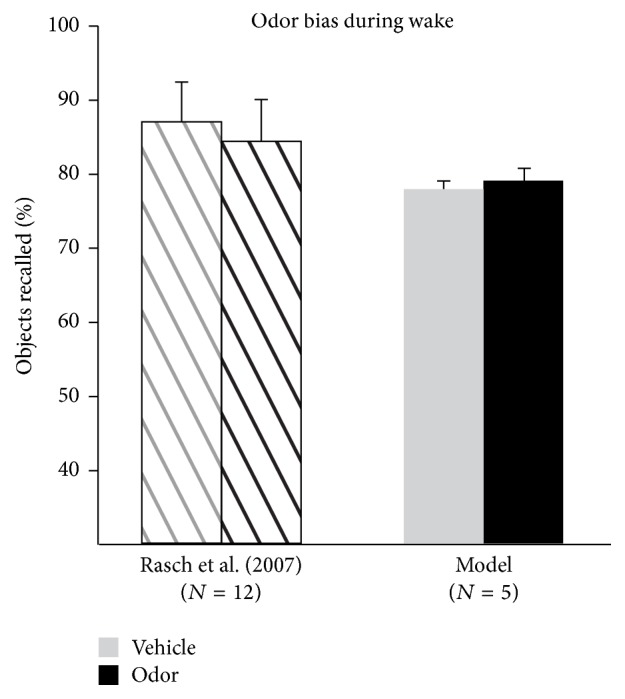
Odor during wake does not bias recall. Experimental data of Rasch et al. (2007) show that presentation of the odor with which Set1 items were learned did not produce differences in recall if the odor was presented during wake (hashed bars). Our model correctly reproduced this data (grey and black bars). Error bars are standard error of the mean.

To further test our model on data and protocols that it was not originally designed for, we conducted simulations aimed at assessing the role of the contextual inputs in targeted memory reactivations during sleep. Experimental work has shown that when items are learned in a specific odor context, presenting that odor during SWS (but not REM) would improve the performance of the subjects during subsequent recall [22] (Figure 9(a) hashed bars). To simulate these experiments, we included a partial representation of the context associated with the learned Set1 within our sleep simulations. Without the addition of partial context inputs to the object-context noise pairs (analogous to the vehicle condition) during replay, our model produced a correct recall fraction comparable to that of REM sleep or SWS-vehicle condition in humans (Figure 9, right most grey bar). A partial presentation of the Set1 contextual inputs (akin to the odor in humans) added to the object-context noise pairs yielded a marked improvement of the recall (Figure 9, black bars). A careful analysis of the manner in which each Set1 item reactivates during sleep in this partial context condition reveals that all memory items are replayed in a similar fashion (Figure 9(b), compared with Figure 7(b)). This result suggests that using context information to bias replay has a strong and uniform effect on the memory engrams associated with that context. During wake, this odor was not found to affect object recall, and we also found no difference in recall if the odor was presented to the model prior to Set1 recall (Figure 10).

## 4. Discussion

In this study, we explored the mechanisms of memory (re)consolidation using a connectionist model of the hippocampus that included segregation into dorsal and ventral pathways and along the proximal-distal CA1 axis, combining object and context information from the LEC and MEC, respectively. Memories are believed to exist as representations of synaptic weights throughout distributed networks using a combinatorial code [60, 61], so that any neuron can contribute to multiple memory item representations. While this property allows a network to store numerous memories, it requires that memories are not entirely separate from one another and must coexist. Computationally, most models implement memories with no representation overlap and while experimenters strive to select stimuli with little to no similarity, they can only hope to approach the zero overlap state used in most models. In this study, we have shown that memory overlap must be included in context-based object reconsolidation to account for the experimental data. Using the model, we quantified the semantic overlap that likely exists in human and rodent studies [9, 10]. Additionally, we investigated the impact of intermediate learning outside of experimentation, a parameter that is difficult for experimenters to control in behavioral studies. Intermediate learning reduced the number of object guesses from Set1 by providing retroactive interference in a way that created competition during memory recall [62]. While this parameter is less crucial to support the behavioral findings, it was a variable that had to be included to properly model this paradigm. Researchers undertaking experimental projects with humans or animals are required to study subjects with preexisting network structures encoding past experiences. Our simulations modeled this constraint by initializing synaptic connection strengths at random. It is the most prudent approach, short of accounting for true subject existing knowledge. The most striking benefit from this implementation is the need for fewer numbers of simulations to recreate experimental findings. Altogether these findings support and partly explain recent evidence that prior knowledge may be crucial for memory reactivation and consolidation [63].

We found that the dorsal CA3 was necessary for context-based object reconsolidation. This result is compatible with previous studies and theories establishing CA3 as the major attractor network of the hippocampus [36, 37]. Note the crucial placement of CA3 in our model, suggesting that CA3 is a “hub” in the hippocampus, mediating object-context associations. Interestingly, when the dorsal CA3 was completely removed, the model recalled Set1 perfectly and without intrusions from Set2. This can be attributed to the fact that, without the dorsal CA3, the network is constrained to learn objects based on the direct LEC to distodorsal CA1 pathway that is isolated from contextual impingement, thus preventing the formation of object-context associations. This suggests that the hippocampus can compose object memories without CA3, but it cannot form object-context associations without it. This may be the “price to pay” for possessing a pattern-completion recurrent network. This relates to animal lesion studies, where rodents without a CA3 were aware of rewards but were impaired at localizing the rewards using contextual clues [64]. Another example of this is found in toddlers; prior to development of CA3 complex object-context associations were unable to be formed using direct LEC to CA1 pathways [65].

Next we evaluated the potential of the trained model to replay memories by introducing noise into the system. Plasticity in network structure enables an animal to adapt to an environment and react appropriately to subsequent identical or similar stimuli. When a network is exposed to a stochastic input, the noise percolates through the learned internal structure to bias the outputs. This is a property of recurrent autoassociative networks that was proposed as a mechanism for memory reactivation [14]. This phenomenon was nicely presented in a human imaging study where learning was shown to sculpt spontaneous brain activity during resting state [66]. Using noise to reactivate memory traces has been done computationally before [51, 52]; however, utilizing these outputs to reprocess memories was absent from these studies. A more recent computational study also showed noise induced memory reprocessing [67], but this model assumed that memories were not strengthened during sleep and instead synaptic connections were downregulated to restore synaptic homeostasis.

Over a decade ago, it was posited that sleep actively downregulates synaptic connections in order to maintain network homeostasis [68, 69]. Since its conception, the sleep and synaptic homeostasis hypothesis has been shown experimentally in the drosophila [70], but more data are lacking. Synaptic homeostasis is an important aspect of normal network functioning; however, it has recently been shown that homeostatic synaptic mechanisms may occur independently of the sleep-wake cycle [71]. Further, a number of studies demonstrated that offline memory processing during sleep is likely additive [18–20] rather than multiplicative as the homeostasis theory implies. Monitoring dendritic branches using two-photon microscopy in live rodents indicated that sleep improved dendritic structural resilience following sleep when memory reactivations occurred [20]. Additionally, it has been shown in humans that sleep protects memories against subsequent interference [18], and we recreated this result by strengthening memories as opposed to using synaptic downregulation. To reconcile these opposing views, a computational study used a spiking Boltzmann network to model sleep by reducing input frequency and plasticity mechanisms and predicted that synaptic homeostasis may occur during REM sleep [72]. This aligns with a recent rodent study demonstrating that animals with disrupted REM sleep show reduced synaptic downscaling compared to control animals [73]. While our simulations did not include downscaling operations per se, our model does modify synaptic weights up and down during learning, and relearning replayed memories may have indirectly acted as a homeostatic mechanism.

Simulating sleep as reactivated memories that are then actively processed matches behavioral data. Compatible with human studies, our findings demonstrated that sleep does not enhance retrieval performance compared to waking in the absence of an interference challenge. We found that memory resilience to interference is significantly improved by learning reactivated memories when compared to not performing additional processing. These results suggest that replayed memories are actively used to process newly formed memories. Molecularly, the genes involved in awake learning are upregulated in sleep [74, 75], further suggesting that additive learning processes occur during sleep. However, this mechanism would suggest that any reexposed memory would be consolidated whether it was correctly stored or not, and indeed it has been shown experimentally that even false memories are enhanced during sleep [76].

Our model suggests that a feedback loop in the hippocampus may be necessary during memory replay. Memory reactivation during sleep in the hippocampus relies on sharp wave ripples, which are thought to originate in CA3 [51, 77] and propagate to the entorhinal cortex [78]. Interestingly memory reactivation in the DG has been shown to follow activity in CA3 [79], which suggests that the signal would have to propagate within the hippocampus in a loop fashion. Experiments have detailed a deep to superficial pathway in the entorhinal cortex that could loop hippocampal output back into the trisynaptic network [80, 81]. Deinhibition of this pathway leads to epileptic seizures [82–84], which suggests that only weak signals circulate naturally. The loop architecture of the hippocampus may allow reactivated memories to be relearned.

The simulations also showed that a separate state of “recall” may not exist in and of itself but that, in fact, recall is always accompanied by learning and subsequent reconsolidation of the items being recalled [3–5]. From this perspective, it is possible that the neural processes underlying the spontaneous firing that occurs during sleep-induced memory replay and the processes that occur during awake memory retrieval could both be the same and could rely on NMDA receptor-mediated spike timing dependent synaptic plasticity [85] (for a computational investigation of memory reconsolidation aiming to understand the role of NMDA and calcium influx in synaptic efficacy, see [86]).

In addition to active learning during sleep, the model shows that partial contextual input can increase reactivation of objects associated with that context. It has been demonstrated that odor exposure during learning in subjects who were awake increased activity in the anterior human hippocampus (ventral in rodents), and odor exposure during slow wave sleep increased activity in both the anterior and posterior hippocampus (ventral and dorsal in rodents, resp.) [22]. This suggests that odor is encoded contextually in the awake state [59], and that its presence in sleep can stimulate the dorsal circuit to reactivate associated objects. Our results reproduce and explain these findings by biasing the stochastic contextual input to the MEC with a partial context which overall increased object reactivation. This suggests that the consolidation of these additional memory reactivations leads to increased recall following sleep. The odor present during sleep allowed weaker stored memories, tied to the association with the odor present during wake, to become reactivated and strengthened.

As in experimental studies, our model showed differences between the effect of reactivation on memory stability in wake or sleep. Wake reactivation degraded memory following a contextual reminder during additional learning, while reactivation during sleep led to enhancement via relearning replayed memories. The difference between these results is rooted in their condition. Wake reactivation occurs in the presence of additional unrelated learning which, in our simulations, is modeled by the learning of a novel object set. In contrast, our model is kept from additional external stimuli during sleep-induced memory reactivation. From this, it appears that memory replay by itself aids in consolidation; however, if reactivation occurs in the presence of additional learning, it leads to a weakening of previous memories and reconsolidation. Additionally, our results demonstrate that odor-driven targeted memory reactivations during sleep can increase consolidation processes, and in wake, this appeared to have no additive effect without the introduction of another learning set. A recent study in humans examined this dichotomy using object value and auditory reminders instead of odor [24].

Learning in our model can be understood as forming attractors in memory space constrained by network structure, and our findings suggest that CA3 is necessary to form a subset of these attractors. By varying the stimulus overlap of context-dependent memories, our results suggest how reactivated attractors interact with newly formed attractors. Additionally, our results also detail how intermediate learning degrades an attractor in memory space. In contrast to degradation, consolidation during sleep appears to stabilize attractors in memory space. Noise propagated through the network can then reactivate memories encoded in synaptic connections, and the context associations defining an attractor can be utilized to bias memory replay during wake and sleep.

## Figures and Tables

**Table 1:** Network parameters. The number of cells is for single sublayers in dorsal/ventral stream and proximal/distal stream. Inhibition is implemented using a *k*-Winner Take All algorithm (*k*-WTA) where *k* is indicated for each layer. Layer compositions are identical for dorsal and ventral divisions as well as proximal and distal.

Layer	Number of cells	*k*-WTA
DG	800	12
CA3	256	6
CA1	400	10

**Table 2:** Model connectivity. Synaptic weights have arbitrary units.

In∖out synaptic strengths	Dorsal DG	Ventral DG	Dorsal CA3	Ventral CA3	Distal dorsal CA1	Proxim. dorsal CA1	Distal ventral CA1	Proxim. ventral CA1
Lateral EC	1	0.3	0.1	0.1	1		0.1	
Medial EC	0.45	1	0.15	0.1		0.15		1
Dorsal DG			1					
Ventral DG				1				
Dorsal CA3			0.1		1	1		
Ventral CA3				0.1			1	1
